# Fathers’ level of involvement in childcare activities and its association with the diet quality of children in Northern Ghana

**DOI:** 10.1017/S1368980022002142

**Published:** 2023-04

**Authors:** Mahama Saaka, Simon Awini, Fred Kizito, Irmgard Hoeschle-Zeledon

**Affiliations:** 1 University for Development Studies, School of Allied Health Sciences, PO Box 1883, Tamale, Ghana; 2 Ghana Health Service, Wa West District Health Directorate, Wechau, Ghana; 3 International Institute of Tropical Agriculture (IITA), Tamale, Ghana; 4 International Institute of Tropical Agriculture (IITA), Ibadan, Nigeria

**Keywords:** Fathers’ involvement, Childcare, Fathers’ nutrition-related knowledge, Fathers’ attitude, Rural settings, Northern Ghana

## Abstract

**Objective:**

This study assessed the level of fathers’ involvement in childcare activities and its association with the diet quality of their children in Northern Ghana.

**Setting:**

The study was carried out in the Northern, Upper East and Upper West regions of Ghana. The people in the study area mostly depend on agriculture as their main occupation.

**Design:**

A community-based comparative analytical cross-sectional study.

**Participants:**

A sample of 422 rural mother–father pairs who had at least one child aged 6–36 months.

**Results:**

The overall level of fathers’ involvement in childcare and feeding activities was high among 63·5 % of the respondents in the 6 months prior to the study. The most common childcare activity men were involved in was providing money for the purchase of food for the child. Minimum acceptable diet was higher for children with a higher level of paternal involvement in childcare activities (adjusted OR = 3·33 (95 % CI: 1·41, 7·90)), compared to their counterparts whose father’s involvement was poor. Fathers who had a positive attitude to childcare and feeding were 2·9 more likely to get involved in childcare activities (adjusted OR = 2·90 (95 % CI: 1·87, 4·48)).

**Conclusions:**

The findings confirm earlier studies that show that fathers’ involvement in childcare activities including feeding is positively associated with improved child feeding practices. The findings point to the need to have a policy shift in which both men and women are key actors in interventions designed to improve child nutritional status in rural settings of Northern Ghana.

Childhood undernutrition associated with poor feeding practices continues to be a public health problem in low- and middle-income countries^(1,2)^ including Ghana. For optimal early childhood growth and development, proper health and nutrition practices are essential. Every child needs an appropriate diet for proper growth and development and yet many households find it difficult to feed their children with appropriate complementary food particularly in low-income countries^(3)^. Suboptimal feeding practices together with infection, poverty and food shortage may be responsible for one-third of childhood malnutrition in poor populations^(4,5)^. Nearly 45 % of deaths among children under 5 years of age are linked to undernutrition mostly in low- and middle-income countries^(6,7)^.

Available evidence suggests improving complementary feeding could reduce child stunting and its related morbidity^(3)^. Appropriate childcare, including adequate feeding and hygiene practices are therefore critical to prevent child undernutrition and impaired development^(8,9)^. Available evidence suggests that traditionally, childcare activities are usually performed by mothers especially in sub-Saharan African countries^(10,11)^. Therefore, over the years, mothers have been the focus of nutrition education, and this has led to a better understanding of nutrition issues among women than their male counterparts. However, when it comes to decision making regarding improving optimal feeding of children, the support of men cannot be over-emphasised. Several studies have shown that household members especially fathers and mothers-in-law exert social influences on a mother’s adoption of optimal infant feeding practices^(12–14)^.

Most community interventions and research seeking to improve the health of women and children target mothers and their children with little attention to fathers as key influencers^(11,15,16)^. This approach of targeting women-only in health and nutrition programming underestimates the influential role of fathers in household decision-making process that directly affects the household ‘s health and nutrition. Some of these decisions a household makes may involve financial choices such as the kind of nutrient-rich foods to buy and how much to spend on foods. It is thus anticipated that involving fathers in childcare and feeding programmes may result in a relative improvement in child nutrition and development. Fathers’ involvement in childcare and feeding practice is a practice wherein fathers actively participate in caring for their children.

Available evidence from sub-Saharan African countries indicates that childcare activities including food preparation and feeding, psychosocial stimulation, hygiene practices and care during illness are most often performed by the mothers as primary care providers^(10,11)^. In particular, paternal involvement in infant and young child feeding decision-making and practices has not been studied adequately in low- and middle-income countries, where household decision making is dominated by men^(17)^. Although several studies have shown a positive association between fathers involvement in childcare activities and optimal child feeding practices^(18–22)^, the evidence is limited and inconclusive. There is, therefore, a dearth of knowledge with regard to the level of male involvement in young child feeding practices and how that is associated with the diet quality of their children in low- and middle-income countries especially in the Ghanaian context. This study therefore assessed the level of fathers’ involvement in childcare activities and its association with the diet quality of their children in Northern Ghana. This study also assessed the predictors of fathers’ involvement in childcare activities.

## Methods

### Study setting

The study was carried out in five districts of Northern Ghana which included Nadowli-Kaleo, Wa West, Tolon/Kumbungu, Savelugu/Nanton and Kassena/Nankana. The West Africa RISING Programme was implemented in these districts. Majority of the people in the study area mostly depend on agriculture as their main occupation while some are involved in trading^(23)^. The main staple foods are maize, sorghum, millet and yam. The rainfall pattern is unimodal and the period is usually short and lasts from May to September, followed by a long dry season (October–April) with dry harmattan winds. Poverty is widespread and the average household per capita daily expenditure is estimated to be $4·91^(24)^.

### Study design, population and sampling

A community-based analytical cross-sectional study was conducted among mother–father pairs and their children aged 6–36 months. The proportion of fathers involved in childcare and feeding activities was not known. So, the sample size was determined on the assumption that 50 % of the targeted fathers would engage in childcare and feeding activities with 5 % margin of error at 95 % confidence level. Based on these assumptions, a sample size of 384 for the study was determined using the Cochran’s sample size formula for categorical data, one-point sample estimation^(25)^:

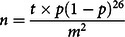

where *n* = required sample size, *t* = confidence level at 95 % (standard value of 1·96), *p* represents the proportion (50·0 %) of fathers involved in childcare activities and *m* = margin of error at 5 % An additional 10·0 % was included to cater for unforeseen circumstances including incomplete and missing questionnaires. The sample size was adjusted to 422 mother–father pairs.

Households were randomly selected from across five districts in Northern Ghana. In each district, five rural communities (clusters) were selected using probability proportionate to size. In each community, twelve couples with at least one child under 3 years of age were selected. The systematic random sampling technique was applied to select eligible couples. In each cluster, a complete list of all households having a married couple was compiled. The total number of households in a cluster was divided by the sample size of 12 to give the sampling interval which guided the selection of the first household. Subsequent households were selected by adding the sampling interval to the previously selected serial number in the sampling frame. This process continued until a minimum of twelve mother–father pairs were selected from a cluster. Only one eligible couple was selected from each household for household interview.

### Study variables and data collection

The primary independent variable was fathers’ involvement in childcare activities. The key outcome measure of interest was minimum acceptable diet (MAD) practice.

In each household, both mother and father were interviewed on different issues. For example, the mother responded to questions regarding infant and young child feeding practices. The fathers responded to questions regarding the level of male involvement in childcare and feeding activities, nutrition-related knowledge and attitude towards child feeding practices. Each team of three members comprising two trained interviewers, and one anthropometrist completed twelve interviews and anthropometric assessments per day. All interviewers could speak fluently the local dialect in which interviews were conducted. The interviews with fathers who were mostly farmers were scheduled for the late afternoon when most of them would have returned from their farms.

The information collected using a structured interview questionnaire included socio-demographic characteristics of fathers and mothers; infant and young child feeding practices; child morbidity and utilisation of health services; level of male involvement in childcare and feeding activities; assessment of fathers’ attitude in child feeding; knowledge of fathers in child feeding and household socio-economic status. A brief description of the key study variables is as follows:

### Level of fathers’ involvement in childcare and feeding activities

The primary independent factor, fathers’ involvement in childcare activities, was quantified based on a modified version used by Thuita and colleagues^(15)^. The level of fathers’ involvement was derived based on responses given where a score of (‘1’) was assigned to (yes) representing the fact that the father played a role and ‘0’ (no) indicating the father did not play a role. The maximum overall score was thus 11. Scores below the median were classified as low fathers’ involvement whilst scores of at least the median were considered to represent high level of fathers’ involvement.

### Measurement of dietary practices

A qualitative 24-hour dietary recall (i.e. detailed information on the types of foods and beverages consumed but no information on quantities) was used to collect information on the child’s diet as reported by the mother^(26)^. The mothers were interviewed by trained field workers about the exact food and beverage intake of their child during the preceding 24 h. Eight food groups including the following were used in the diet recall: (i) grains, roots and tubers; (ii) legumes and nuts; (iii) dairy products; (iv) flesh foods (meat, poultry and fish) (v) eggs; (vi) vitamin A-rich fruits and vegetables; (vii) other fruits and vegetables and (viii) breastmilk.

The indicators of minimum meal frequency, minimum dietary diversity and MAD were used for assessing child feeding practices as per WHO guidelines^(27)^.

The minimum meal frequency indicates whether a child has taken ‘adequate number of meals in the past 24 h. Adequacy of meals taken means a child had received the minimum frequency for appropriate complementary feeding (that is, two times for 6–8 months and three times for 9–11 months, three times for children aged 12–23 months) in last 24 h. For non-breast-fed children, the minimum meal frequency was 4. A child aged 6–23 months who has eaten from at least five food groups out of eight food groups in the past 24 h is deemed to have met minimum dietary diversity ^(27)^. A child who met both minimum meal frequency and minimum dietary diversity was classified as being fed a MAD. Appropriate complementary feeding practice was measured using a composite index comprising scores of minimum meal frequency + minimum dietary diversity + timely introduction of complementary foods at 6 months. The summative score for appropriate complementary feeding practice was thus classified as appropriate if the mother practiced all the above three indicators, as recommended and inappropriate if at least one indicator was not met.

To assess the timely introduction of complementary foods, mothers/caretakers were asked two closed-ended questions as follows: Is child currently eating other foods apart from breast milk? If yes, when did you start complementary feeding?

### Assessment of nutrition-related knowledge

Nutrition-related knowledge was assessed as per the FAO guidelines^(28)^. Knowledge was defined as the father’s ability to remember and correctly recall basic food and nutrition information and/skills in complementary feeding. The total knowledge score for each respondent was based on correct answers to twenty nutrition knowledge-related questions. The scores were categorised as high if total correct responses were > 70 %, and scores ≤ 70 % were classified as low.

### Assessment of fathers’ attitude in child feeding practices

The nutrition-related attitudes were derived from assessing eleven key positive and negative behaviours/statements which were formulated and pretested by the principal researcher. These statements are based on common parameters related to appropriate child feeding. Attitude was measured on a three-point Likert scale with three response options (agree, neutral, disagree), details of which are provided in a supplementary file no 1.

A score of 0 was given to disagree responses while a score of 1 was assigned to being neutral and 2 for agreeing to positive attitude. A summative attitude score for each respondent was therefore obtained. Scores of respondents were categorised as positive if total correct responses were ≥ median score, otherwise it was regarded as negative. The overall knowledge, attitude and practices score was quantified by adding the individual score of knowledge, attitude and practices. Respondents were categorised as having high score if total correct responses were ≥ median score. Scores less than the median were regarded as low.

### Measurement of household wealth index

Household wealth index, proxy indicator for socio-economic status of households was quantified using the principal component analysis. Variables included in the principal component analysis were household assets and housing quality (floor, walls and roof material), source of drinking water, type of toilet facility, the presence of electricity, type of cooking fuel and ownership of modern household durable goods and livestock (e.g. bicycle, television, radio, motorcycle, sewing machine, telephone, cars, refrigerator, mattress, bed, computer and mobile phone)^(29–32)^.

### Data analysis

The Statistical Package for the Social Sciences, version 23.0 (SPSS) was used to perform the statistical analyses. The complex survey module was used to consider the complex design of cluster sampling. This allowed for calculating valid standard errors from the sample data, thereby making correct population inferences. Bivariate analysis was conducted using the chi-square test statistic to assess the association between categorical variables. Using forward methods, multivariate logistic regression models were used to determine associations between independent variables and the main outcome (MAD). Statistical significance was determined at the p-value less than 0·05. Adjusted OR and their 95 % CI were reported.

## Results

### Socio-demographic characteristics of study participants

The mean age for fathers was 36·5 ± 9·2 years. Most mothers (56·4 %) had no formal education and 56·9 % of households had high wealth index. Fathers were mostly Dagomba and 52·4 % of them were Muslims. Most of the households (73·5 %) had one child who were under 2 years (Table 1).

**Table 1:** Socio-demographic characteristics of sample

Characteristics	Frequency (*n*)	Percentage (%)
Father’s age (years)		
Under 30 years	89	21·1
30–39	191	45·3
40–49	111	26·3
At least 50 years	31	7·3
Educational level of mothers		
None	238	56·4
Primary	78	18·5
Middle/junior high school	55	13·0
Senior high school/vocational training	38	9·0
Tertiary	13	3·1
Ethnicity		
Dagomba	168	39·8
Kasena	61	14·5
Wala	58	13·7
Dagaaba	110	26·1
Frafra/Builsa/Kusasi	10	2·4
Nankam	15	3·5
Religion		
Christianity	201	47·6
Islam	221	52·4
Classification of household wealth index		
Low (Less than median score of 8)	182	43·1
High (At least median score of 8)	240	56·9
Number of children		
1	310	73·5
2	92	21·8
>2	20	4·7

### Level of fathers’ involvement in childcare and feeding activities

The overall level of male involvement was high among 63·5 % of the respondents in the 6 months prior to the study (Table 2). The most common childcare and feeding activities men were involved in were providing money to buy food for the child, providing money for transport to child health clinic and assisting in household chores like handling and or playing with the child. The least activity men were involved in was assisting in household chores of sweeping the compound.

**Table 2:** Responses to questions/statements used to assess the level of fathers’ involvement in childcare activities in the past 6 months (multiple responses possible)

Activity	Response	
Health and finance	Frequency (*n*)	Percentage (%)
Have you ever taken your child to health institution since his birth?	254	60·2
Provide money to buy food for the child	404	95·7
Provide transport money to child health clinic	392	92·9
Assisting with childcare and household level activities		
Participate in child feeding (e.g. father feeding the child and deciding the ‘kind of foods’ the child eats)	337	79·9
Bathing the child	253	60·0
Assist in fetching water	214	50·7
Helps with cooking	169	40·0
Handling and or playing with the child	386	91·5
Sweeping the compound	159	37·7
Accompanies wife/attends growth monitoring sessions	191	45·3
Accompanies wife/attends child’s other medical consultations	322	76·3
Categorisation of male involvement		
Low (less than median score)	154	36·5
High (at least the median score)	268	63·5

### Factors associated with fathers’ involvement in childcare activities

The factors which associated with fathers’ involvement in childcare activities are shown in Table 3. The key influencing factors identified were child’s age, father’s nutrition-related attitudes, district of residence and the socio-economic status of the household as measured by household wealth index.

**Table 3:** Factors associated with fathers’ involvement in childcare activities (Multivariable logistic regression analysis)

		95 % CI
Factors	Adjusted OR	Lower, Upper
Classification of child’s age (months)		
6–11 months	Reference	Reference
12–23	1·76	1·12, 2·77
24–36	3·04	1·40, 6·58
Household wealth index		
Low	Reference	Reference
High	1·69	1·09, 2·62
Nutrition-related attitudes score		
Negative	Reference	Reference
Positive	2·85	1·78, 4·58
District of residence		
Wa West	Reference	Reference
Savelugu	2·39	1·19, 4·79
Tolon	0·64	0·32, 1·28
Kassena/Nankana	2·27	1·17, 4·39
Nadowli/Kaleo	1·80	0·92, 3·50

In a multivariable logistic regression analysis, fathers who had a positive attitude to childcare and feeding were 2·9 times more likely to get involved in childcare and feeding practices AOR = 2·85 (95 % CI: 1·78, 4·68). Fathers of high socio-economic status as measured by household wealth index were also 1·7 times more likely to be involved, compared to their counterparts who were poor AOR = 1·69 (95 % CI: 1·09, 2·62). Paternal involvement in childcare and feeding was significantly higher among older children (24–36 months), compared to younger children AOR = 3·04 (95 % CI: 1·40, 6·58). Fathers who were residents in the Savelugu District had a higher level of involvement, compared to fathers who were residents in the Wa West District AOR = 2·39 (95 % CI: 1·19, 4·79).

### Predictors of minimum acceptable diet

After controlling for potential confounding factors in a multivariable logistic regression analysis, father’s involvement in childcare activities remained a significant independent predictor of MAD (Table 4). Children whose fathers’ involvement in childcare and feeding activities was high were 3·3 times more likely to be fed a MAD (AOR = 3·33 (95 % CI: 1·41, 7·90)), compared with their counterparts whose fathers’ involvement had been low. Children whose fathers had high nutritional knowledge were 1·7 times more likely to be fed a MAD (AOR = 1·65 (95 % CI: 1·08, 2·50)), compared with their counterparts whose fathers had low nutritional knowledge. Compared with women whose husbands did not participate in care group nutrition education, women whose husbands took part in care group nutrition education sessions were 2·3 times more likely to feed their children a MAD (AOR = 2·26, (95 % CI: 1·41, 3·60)). Women who had a higher educational level of at least Senior High School were 2·1 times more likely to feed their children a MAD (AOR 2·05; 95 % CI (1·05, 3·98)), compared to their counterparts who had no formal education. A unit increase in house wealth index was associated with 1·1 increase in MAD (AOR 1·09; 95 % CI (1·01, 1·16)). The set of predictors accounted for 12·3 % of the variation in MAD (Nagelkerke R Square = 0·123).

**Table 4:** Predictors of minimum acceptable diet (Multivariable logistic regression analysis)

		95 % CI
Predictor	Adjusted OR	Lower, Upper
High fathers’ nutrition knowledge	1·65	1·08, 2·50
High fathers’ involvement in childcare activities	3·33	1·41, 7·90
Household wealth index	1·09	1·01, 1·16
Mother’s educational level		
None	Reference	Reference
Low (primary and junior high school)	1·40	0·89, 2·21
High (at least senior high school)	2·05	1·05, 3·98
Care group member?		
No	Reference	Reference
Yes	2·26	1·41, 3·60

## Discussion of results

To the best of our knowledge, this study is the first to investigate fathers’ involvement in childcare activities and its association with diet quality of children aged 6–36 months in rural households of Northern Ghana. This study also assessed the predictors of fathers’ involvement in childcare activities. The overall level of fathers’ involvement in childcare activities in the 6 months prior to the study was 63·5 %.

### Level of fathers’ involvement in childcare and feeding activities

The results of this study showed that a high proportion of fathers were involved in childcare activities and this is consistent with other studies from African countries^(10,33)^, where fathers reportedly took part in feeding, cooking, keeping companionship with the child or cleaning the child especially when the mothers were away from home. The involvement of fathers in childcare is also reported in a recent study conducted in an urban slum in Bangalore, India^(34)^. Paternal involvement in childcare and related activities across different regions of the world has been measured variously and the level of engagement varies according to local circumstances including the cultural environment^(35–37)^. In Ethiopia, fathers were found to be supportive of breastfeeding practices^(38)^.

The results also showed that father’s involvement in childcare activities was mostly through the provision of money to purchase food and transport money to child health clinic. This finding is consistent with several other studies which reported that men placed more importance to their economic contribution to the household or bread winner’ role than they did in other ways of direct involvement in child care and feeding activities^(10,15,19,39)^. Assisting in household chores especially sweeping the compound, helping with cooking and accompaniment to growth monitoring sessions were less common than financial support. This is perhaps culturally; men are perceived as providers and controllers of resources in most households and not caretakers^(11,19,33)^. Barriers such as lack of support from fellow men may be contributing to this low involvement in providing physical support. Men who are involved in direct child-feeding practices may be regarded by colleagues as being influenced by their wives. Traditional gender roles expect men to be providers of material and financial support while women are more aligned to household chores as part of their responsibilities.

To reduce the burden of malnutrition, the need for greater men ‘s involvement in child care and feeding practices cannot be over-emphasised^(40)^. Unfortunately, male involvement has largely been overlooked as nutrition programmes focus largely on pregnant and lactating women^(41)^. It is therefore critical that more approaches to involve men should receive priority and health services programming. A drive for a gradual change in men’s attitude, coupled with men serving as role models for others can be a source of motivation to change.

### Relationship between fathers’ involvement in childcare activities and child feeding practices

This study assessed the relationship of fathers’ involvement in childcare activities and child feeding practices reported by their mothers. The results showed a significant positive association between the overall male involvement in childcare activities and MAD in the past 24 h. Children whose fathers’ involvement in childcare and feeding activities was high were 3·3 times more likely to be fed a MAD. The findings are consistent with earlier studies conducted elsewhere which indicated the need to involve fathers in nutrition interventions including complementary feeding practices. For example, a study in Ethiopia found that male involvement increased dietary diversity in the household by 13·7 %^(42)^. Child-feeding surveys in Kenya have also shown that involving fathers at the community, household and individual level improved child-feeding practices in the long term^(43)^. Furthermore, a comparative analysis of complementary feeding in Bangladesh, Malawi, Peru and Zambia showed that the targeting of fathers with messages of nutrition was an important step towards behavior change to optimal complementary feeding practices of their children^(44)^. Nutrition education sessions targeted at fathers improved child-feeding practices in Kenya, Ethiopia and Vietnam^(13,20,45)^.

### Factors influencing fathers’ involvement in young child feeding practices

Infant and young child feeding is key to promoting healthy growth and development, particularly during the first two years of a child’s life. In this study, socio-economic status was an important predictor of fathers’ involvement in childcare and feeding activities. A study in Bangalore has also noted that per capita income was independently associated with poor involvement of fathers in child feeding^(34)^. Traditionally, fathers are expected to contribute financially to the family^(46)^. The consequence of this is that low-income fathers are likely to be poorly involved in child care and feeding since they may not have a lot of money to provide for the child’s feeding needs^(47,48)^. Gavin *et al.* ^(47)^ noted that fathers’ involvement is often negatively impacted due to the father’s inability to provide financially. Having a sense of inadequacy in their contributions to their family, low-income men are often discouraged from being involved in other areas of care^(48)^. This emphasis on fathers’ financial contribution to child care can also discourage more direct involvement from fathers because men who provide a steady income for the family may feel that their most important role has been accomplished^(49)^.

Male involvement was also limited by negative attitude towards child-feeding practices. With a positive attitude to childcare activities, the level of fathers’ involvement in child feeding increased and this association has been reported in other studies^(50,51)^.

Fathers’ involvement in childcare and feeding was significantly higher among older children (24–36 months), compared to younger children. It appears fathers’ involvement in childcare and feeding practices increases as the child grows older and begins to eat other foods other than breastmilk. This finding concurs with other studies have reported that fathers’ care toward child feeding becomes active beyond the age of 2 years^(15)^.

### Strengths and limitations of the study

One of the strengths of this study is that it is one of the few studies that have assessed fathers’ involvement in childcare activities and its relationship with dietary intake of children. However, the study has some limitations including the fact that the study design was cross-sectional which has the inherent inability to establish causality. There is also a possibility of recall biases which are inevitable in self-reported feeding practices. This might have led to measurement bias, although we do not expect this bias to be differential, and thus we do not expect that the observed association between fathers’ involvement in childcare activities and MAD would be changed.

## Conclusion

The involvement of men in childcare activities was high particularly regarding financial support for food and health care in rural settings of Northern Ghana. Assisting in household chores especially sweeping the compound, helping with cooking and accompaniment to growth monitoring sessions were less common than financial support. Our findings confirm earlier studies that show that fathers’ involvement in childcare activities including feeding is positively associated with improved child-feeding practices. The findings point to the need to have a policy shift in which both men and women are key actors in interventions designed to improve child nutritional status in rural settings of Northern Ghana.

## Supplementary material

For supplementary material accompanying this paper visit https://doi.org/10.1017/S1368980022002142. click here to view supplementary material
