# Supplementary material for: Fathers’ level of involvement in childcare activities and its association with the diet quality of children in Northern Ghana
Source: Public Health Nutr. 2022 Oct 10;26(4):771–8. doi: 10.1017/S1368980022002142 (PMC10131150; doi:10.1017/S1368980022002142)
Supplement: Supplementary file 1 [file S1368980022002142sup001.docx]

**Supplementary File 1: Questionnaire for the assessment of nutrition-related attitudes towards child feeding**

I would like to mention some key childcare and feeding practices. Please tell me how much you agree with them.

| **Variable** | **How much do you agree with the message?**  **1.Agree 2. Do not know (neutral) 3. Disagree** |
| --- | --- |
| 1. It is important to breastfeed a child within one hour after birth |  |
| 1. Breastfeeding for 2 or more years is good |  |
| 1. “Introduction of complementary foods to children should commence at six months |  |
| 1. A child aged 9-23 months eat at least 3 times in a day |  |
| 1. “Give children a variety of foods for healthy growth” |  |
| 1. “Green leafy vegetables help the body to make blood for children and adults” |  |
| 1. “Give your children fish and meat frequently and in sufficient amounts” |  |
| 1. “Add fats and oils to your child’s food for energy and vitality” |  |
| 1. “Give thick porridge to children for better nutrition and growth” |  |
| 1. “Increase the consistency of your child’s foods as he/she gets older” |  |
| 1. “Always remember to wash your hands with soap before handling food for the family” |  |

**QUESTIONNAIRE ON FATHES’ NUTRITION RELATED KNOWLEDGE IN YOUNG CHILD FEEDING PRACTICES**

1. If a woman gives birth to a new-born, how long should it take for the baby to start breastfeeding?
2. Within first hour of delivery
3. 2 to 23 hours after delivery
4. The next day (more than 24 hours)
5. Do not remember

2.What is the best food for a new-born baby?

1. Only breastmilk
2. Other
3. Don’t know

3.What is the recommended minimum number of years that a woman should breastfeeds her child?

1. Six months or less
2. 6–11 months
3. 12–23 months
4. 24 months and more
5. Don’t know

4. At what age should babies start eating foods in addition to breastmilk?

1. At six months
2. Don’t know
3. Other (Specify)……………………………………………….

5. How many times should a child aged 9-23 months eat in a day?

1. Once
2. Two times
3. At least 3 times
4. Do not know

6. It is recommended that a woman waits at least two or three years between pregnancies, that is before becoming pregnant once again. Mention one reason why this is recommended?

1. To rebuild/fill up their body stores of nutrients (fat, iron and others)
2. For the mother to be healthier before having a new baby/to be prepared for the arrival of a new baby
3. Don’t know
4. Why do you think children should eat variety of food groups, including fruits and vegetables, protein sources, dairy products and grains? (Multiple responses possible)
5. Protection against diseases
6. Promote growth and development
7. Gives blood/energy
8. Other (Specify) …………………………………………….
9. Cannot tell.

8. What should a lactating mother do to have enough breastmilk?

1. Eat plenty food such as hot porridge
2. Frequent breast feeding,
3. Correct positioning and good attachment to the breast.
4. Cannot tell

**9.** Give one reason why green leafy vegetables should be consumed by both children and adults.

1. Protection against diseases
2. Promote growth and development
3. Supply minerals and vitamins/Gives blood
4. Gives energy
5. Cannot tell

10. If a child eats TZ+ Okro soup and another child eats rice + beans stew, which of them is likely to have more blood?

11. If the chief of your community told you that “A young child (aged 6 up to 24 months) should not be given animal foods such as eggs and meat”. What will you tell him?

1. The chief is wrong because such foods are needed for the growth of the child.
2. The chief is right because eating such foods makes the child to become thief in the future
3. The chief is right because he is the chief and knows what is good for the child
4. Does not know what to tell the chief

12. Which of the following foods can give more blood?

1. Beans
2. Liver
3. Green leafy vegetables
4. Cannot tell

13.Mention three reasons why you think it is important to give foods in addition to breastmilk to babies from the age of six months. (Multiple responses possible)

1. Breast milk alone is inadequate at age 6 months
2. Provide more nutrients for growth
3. Provide more blood for the child
4. Cannot mention

14. Health workers recommend that children less than 6 months should not be given water. Give three good reasons to support this. (Multiple responses possible)

1. To avoid infection
2. Water will occupy the stomach and therefore prevent intake more breastmilk
3. Breastmilk itself has enough water for the child
4. Don’t know
5. Other (specify) ……………………………………………………………………

15. Could you please, mention **3 ways** to encourage young children to eat well? (Multiple responses possible)

1. Giving them attention during meals, talk to them, make mealtimes happy times
2. Clap hands
3. Make funny faces/play/laugh
4. Demonstrate opening your own mouth very wide/modelling how to eat
5. Say encouraging words
6. Draw the child’s attention
7. Serve the preferred food of the child
8. Do not know

16. All the foods in this world can be grouped into four. Mention three of such groups (Multiple responses possible)

1. Energy giving foods, e.g. maize, yam
2. Animal source foods
3. Plant-based protein foods e.g. legumes such beans, groundnuts, nieri, agushie
4. Protective food such as fruits and vegetables, e.g. bean and cassava leaves, mango, pawpaw. Protective foods are rich in minerals and vitamins and they protect the body from infections and strengthen the immune system.

17. Mention three reasons why some children are not fed well by their mothers. (Multiple responses possible)

1. Lack of food
2. Poverty
3. Lack of nutrition knowledge
4. Cultural barriers etc.
5. Cannot mention

18. Mention three consequences of malnutrition in pregnant women that affects birth outcomes. (Multiple responses possible)

1. Increased risk of fetal neonatal and infant death
2. Intra‐ uterine growth restriction
3. Low birth weight and prematurity
4. Birth defects
5. Cretinism
6. Brain damage
7. Increased risk of infection

19. Give three reasons why mothers or caregiver should give the child soft foods or thick porridge that have been enriched with milk, oil, groundnuts paste, peas or beans, egg or sugar to make it more nutritious. (Multiple responses possible)

1. Thin watery gruels are not healthy for the baby as they fill the stomach without providing enough energy and nutrients.
2. There is no single food that has all the nutrients the body needs and so the child must eat different foods.
3. The child's stomach is small and so cannot take large amounts of food at a go. Therefore, feed your baby **frequently** with **small amounts** of thick porridges so that she/he grows strong and healthy.
4. Do not know
5. Other (specify) ………………………………………………………………………….

20. Suggest three ways men can help their wives to improve nutrition in the family.

(Multiple responses possible)

1. Men can support their wives to access diverse diets during and after pregnancy (e.g. providing money for food ingredients like meat, fish eggs).
2. Men can help improve women's nutrition by helping them with their workload such as fetching water, helping with childcare or sweeping the compound.
3. Including men in nutrition education activities and discussions can help them recognize the importance of providing nutritious food to their families and increasing their participation in household chores related to childcare and feeding.
4. Men can also take up a supportive role in agricultural activities, providing women with productive farming land.
